# Effect of Repetitive Passive Movement Before Motor Skill Training on Corticospinal Excitability and Motor Learning Depend on BDNF Polymorphisms

**DOI:** 10.3389/fnhum.2021.621358

**Published:** 2021-02-05

**Authors:** Manh Van Pham, Shota Miyaguchi, Hiraku Watanabe, Kei Saito, Naofumi Otsuru, Hideaki Onishi

**Affiliations:** ^1^Institute for Human Movement and Medical Sciences, Niigata University of Health and Welfare, Niigata, Japan; ^2^Graduate School, Niigata University of Health and Welfare, Niigata, Japan; ^3^Department of Physical Therapy, Hai Duong Medical Technical University, Hai Duong, Vietnam; ^4^Department of Physical Therapy, Niigata University of Health and Welfare, Niigata, Japan

**Keywords:** repetitive passive movement, motor learning, visual tracking task, transcranial magnetic stimulation, motor evoked potential, primary motor cortex, homeostatic plasticity, brain-derived neurotrophic factor

## Abstract

A decrease in cortical excitability tends to be easily followed by an increase induced by external stimuli via a mechanism aimed at restoring it; this phenomenon is called “homeostatic plasticity.” In recent years, although intervention methods aimed at promoting motor learning using this phenomenon have been studied, an optimal intervention method has not been established. In the present study, we examined whether subsequent motor learning can be promoted further by a repetitive passive movement, which reduces the excitability of the primary motor cortex (M1) before motor learning tasks. We also examined the relationship between motor learning and the brain-derived neurotrophic factor. Forty healthy subjects (Val/Val genotype, 17 subjects; Met carrier genotype, 23 subjects) participated. Subjects were divided into two groups of 20 individuals each. The first group was assigned to perform the motor learning task after an intervention consisting in the passive adduction–abduction movement of the right index finger at 5 Hz for 10 min (RPM condition), while the second group was assigned to perform the task without the passive movement (control condition). The motor learning task consisted in the visual tracking of the right index finger. The results showed that the corticospinal excitability was transiently reduced after the passive movement in the RPM condition, whereas it was increased to the level detected in the control condition after the motor learning task. Furthermore, the motor learning ability was decreased immediately after the passive movement; however, the motor performance finally improved to the level observed in the control condition. In individuals carrying the Val/Val genotype, higher motor learning was also found to be related to the more remarkable changes in corticospinal excitability caused by the RPM condition. This study revealed that the implementation of a passive movement before a motor learning tasks did not affect M1 excitatory changes and motor learning efficiency; in contrast, in subjects carrying the Val/Val polymorphism, the more significant excitatory changes in the M1 induced by the passive movement and motor learning task led to the improvement of motor learning efficiency. Our results also suggest that homeostatic plasticity occurring in the M1 is involved in this improvement.

## Introduction

Neuroplasticity, the supposed mechanism underlying memory and learning, is an important neurophysiological phenomenon that is also related to motor learning and functional recovery in patients with stroke (Hosp and Luft, 2011). When the excitability of the neuronal population is reduced, neurons can easily increase it by external stimuli via a mechanism aimed at recovering their excitability; this process is called “homeostatic plasticity” (Turrigiano, 2011). It has been reported that the improvement of neuroplasticity and motor skills can be promoted using homeostatic plasticity (Ziemann et al., 2004; Ziemann and Siebner, 2008; Jung and Ziemann, 2009). Repetitive transcranial magnetic stimulation (rTMS) and transcranial direct current stimulation (tDCS) are non-invasive brain stimulation methods that can be used to improve motor learning ability (Muellbacher et al., 2002; Nitsche et al., 2003). rTMS increases the excitability of the primary motor cortex (M1) at a frequency ≥5 Hz, while it decreases this parameter at a frequency ≤1 Hz (Pascual-Leone et al., 1994; Chen et al., 1997). Moreover, the excitability of the M1 can be increased by anodal-tDCS to the M1, whereas it can be decreased by cathodal-tDCS (Nitsche and Paulus, 2000, 2001). A previous study that combined these two intervention methods reported that M1 excitability was increased by rTMS at 5 Hz after the cathodal-tDCS intervention, whereas it was decreased by rTMS at 5 Hz after the anodal-tDCS intervention (Lang et al., 2004). Therefore, this phenomenon seems to be related to homeostatic plasticity, because the effect of rTMS intervention on the M1 depends on the excitability of this brain structure before the intervention.

Post-exercise depression (PED) consists in the decrease in corticospinal excitability after low-load repetitive voluntary movements (Brasil-Neto et al., 1994; Teo et al., 2012a; Miyaguchi et al., 2016). PED occurs after repetitive passive movement (RPM), as well as after voluntary movements (Miyaguchi et al., 2013; Sasaki et al., 2017; Onishi, 2018) Moreover, M1 excitability has been transiently decreased via an RPM intervention for 10 min (Miyaguchi et al., 2013; Sasaki et al., 2017; Onishi, 2018). PED is supposed to be a phenomenon occurring within the M1 because the F wave, which is an index of spinal cord excitability, does not change after passive movement (Sasaki et al., 2017; Onishi, 2018), whereas short interval intracortical inhibition (SICI), which is an index of the excitability of the GABAergic inhibitory circuits within the M1, increases after passive movement (Sasaki et al., 2017; Onishi, 2018). Jung and Ziemann (2009) also reported that increased M1 excitability prior to motor learning decreased subsequent motor learning ability and vice versa (Jung and Ziemann, 2009). Thus, we hypothesized that, when performing a motor learning task during the PED caused by passive movement, homeostatic-plasticity-like plastic changes also occur in the M1, thereby improving the motor learning ability. Therefore, if passive movement, which is widely used in clinical practice, can induce homeostatic-plasticity-like changes and improve motor learning ability, it may be used as a highly versatile motor program.

The brain-derived neurotrophic factor (BDNF), which is a nerve growth factor, is a protein that plays an important role in synaptic development and growth, as well as in the regulation and plastic changes of GABAergic synaptic transmission and glutamatergic neurotransmission (Wardle and Poo, 2003; Jovanovic et al., 2004; Baldelli et al., 2005; Carvalho et al., 2008). Previous studies have reported that BDNF is related to plastic changes in the M1 (Kleim et al., 2006; Antal et al., 2010; Lee et al., 2013) and to motor learning (Vaynman et al., 2004; Fritsch et al., 2010; McHughen et al., 2010). The mutant form of the *BDNF*, in which the 196th base is changed from G to A, resulting in mutation of the 66th amino acid of BDNF from valine (Val) to methionine (Met). It is known that the secretory function of BDNF in the brain is decreased by the change of Val to Met (Egan et al., 2003). In recent years, it has become clear that differences in BDNF polymorphisms affect neural plasticity, and thus the recovery of stroke patients and its mechanisms are also affected by BDNF polymorphisms (Di Pino et al., 2016). Moreover, it has recently been reported that BDNF polymorphisms are related with homeostatic plasticity, a phenomenon that is unlikely to occur in carriers of mutant (Val/Met and Met/Met) vs. wild-type (Val/Val) genotypes (Cheeran et al., 2008).

Thus, the present study aimed to elucidate whether corticospinal excitability and motor learning ability are improved after the performance of motor learning tasks during the PED caused by passive movement, and whether the *BDNF* gene polymorphisms contribute to these effects.

## Methods

### Subjects

The participants of the present study were 40 healthy adult students [22 males; age (mean ± standard deviation (range)), 21.9 ± 1.8 (20–32) years; Val/Val genotype: 17 subjects; Met carriers, 23 subjects] at Niigata University of Health and Welfare. The subjects were randomly assigned to one of two groups (20 subjects in each group). The first group was subjected to the 5 Hz repetitive passive movement intervention condition (RPM condition; 10 males, aged 21.6 ± 0.9 (20–23) years; Val/Val genotype: 9 subjects; Met carriers, 11 subjects), whereas the second group was subjected to the control condition [Control condition; 20 subjects (12 males), aged 22.3 ± 2.3 (21–32) years; Val/Val genotype: 8 subjects; Met carriers, 12 subjects]. All participants were right-handed, were not taking any medication, and had no central nervous system disease, psychiatric disorder, or orthopedic disease. The study followed the recommendations of the ethics committee of Niigata University of Health and Welfare, who approved the protocol, and was conducted in accordance with the principles of the Declaration of Helsinki. The recruitment of subjects was conducted in accordance with the ethical rules of the Ethics Committee of Niigata University of Health and Welfare. In addition, written informed consent was obtained from all subjects.

### Electromyography (EMG) Measurement

The EMG measurements targeted the right first dorsal interosseous (FDI) muscle, which was monitored using disposable Ag/AgCl electrodes in a belly-tendon montage. The earth electrode was wrapped around the right forearm. The EMG signals were amplified by an amplifier (A-DL-720140, 4 Assist, Tokyo, Japan), processed by an A/D converter (Power Lab, AD Instruments, Colorado, USA) at a sampling frequency of 4 kHz, and then stored on a computer. For EMG analysis, a 20 Hz high-pass filter was employed together with a biological signal analysis software (Lab Chart 7; AD Instruments, Sydney, Australia).

### Transcranial Magnetic Stimulation

Transcranial magnetic stimulation (TMS) was delivered through a figure eight coil (diameter, 9.5 cm) that was connected to a Magstim 200 stimulator (Magstim, Dyfed, UK). The coil was held tangentially to the skull over the left primary motor cortex (M1) with the handle pointing posterolaterally at 45° to the sagittal plane in the position producing the largest motor evoked potential (MEP) from the right FDI muscle. The position and orientation of the coil were marked by magnetic resonance imaging (MRI) using the Visor2 TMS neuronavigation system (eemagine Medical Imaging Solutions GmbH, Berlin, Germany), and held in place. T1-weighted images were obtained using a 1.5 T MRI scanner (SIGNA HD, GE Healthcare, Milwaukee, WI, USA) before initiating the experiment. The TMS intensity was set to evoke an MEP of ~1 mV in the right FDI muscle. Consistent with previous studies (Miyaguchi et al., 2017; Rawji et al., 2018), TMS was delivered at a rate of 0.2 Hz during data collection, which was performed 15 times at rest (Vaseghi et al., 2015; Tsuiki et al., 2019).

### Repetitive Passive Movement (RPM) Task

**Figure 1:**
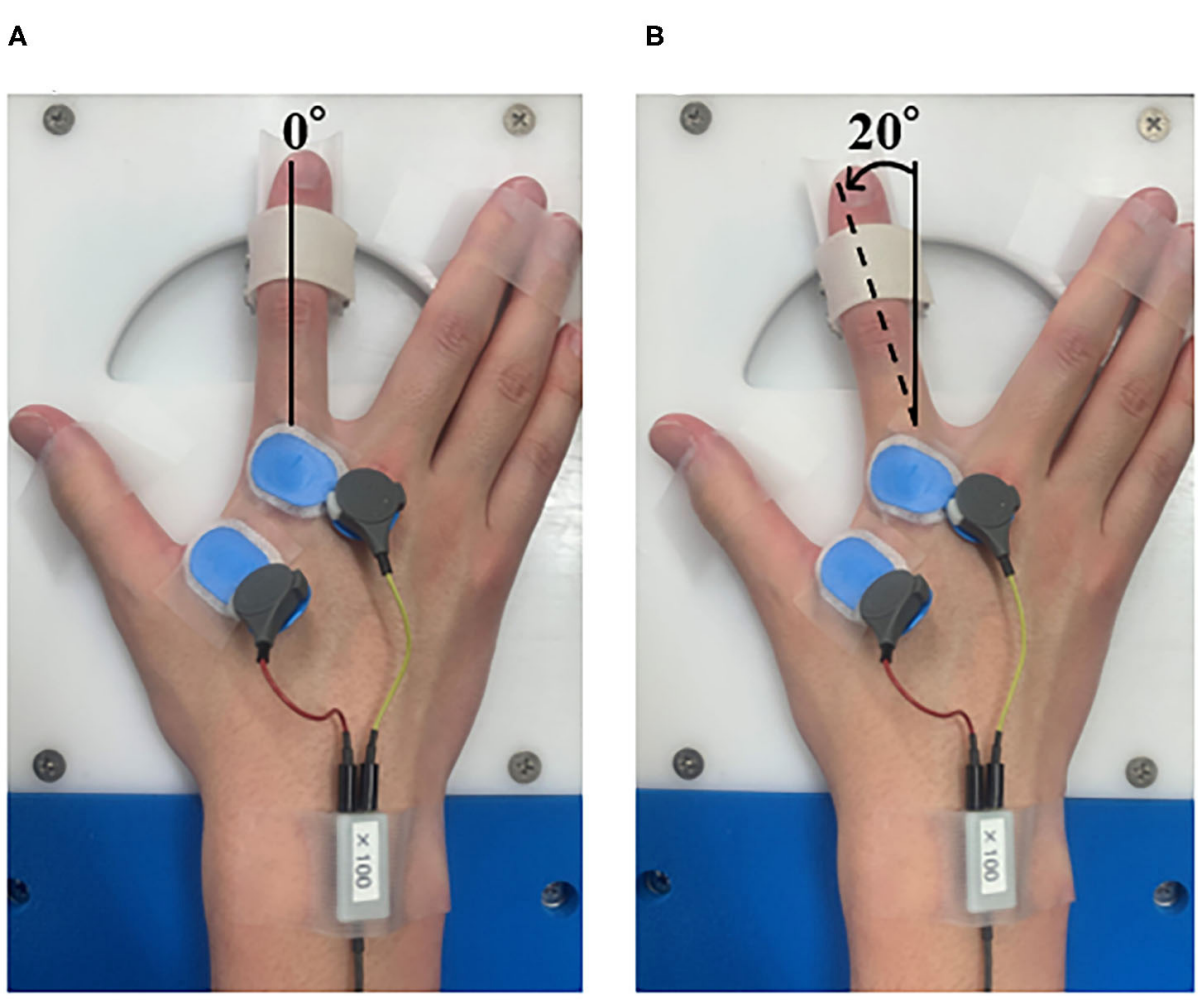
Passive movement intervention. **(A)** Abduction of the metatarsophalangeal (MP) joint at 0°. **(B)** Abduction of the metatarsophalangeal (MP) joint from 0 to 20°. In the RPM condition, the right index finger was moved passively for 10 min. The angular velocity was 40°/s, the movement frequency was 5 Hz, and the movement range was 0–20° abduction of the metatarsophalangeal (MP) joint.

The preconditioning task administered before the motor learning task used repetitive abduction–adduction of the right index finger (Figure 1). We employed a custom RPM control device (Takei Kiki Kogyo, Niigata, Japan) to control the speed and angle of movement, as described previously (Sasaki et al., 2017; Tsuiki et al., 2019). We set the intervention time to 10 min. The passive movement was repeated continuously with an angular velocity of 40°/s and a movement frequency of 5 Hz in the RPM condition. We set the range of movement of the index finger from 0° to 20° for the abduction of the metacarpophalangeal (MP) joint, and the position of 0° at the midpoint of the MP joint. In a similar position as that used for the RPM condition, the control condition fixed the index finger to the RPM control device, to maintain the resting sitting position for 10 min.

### Motor Performance Task

**Figure 2:**
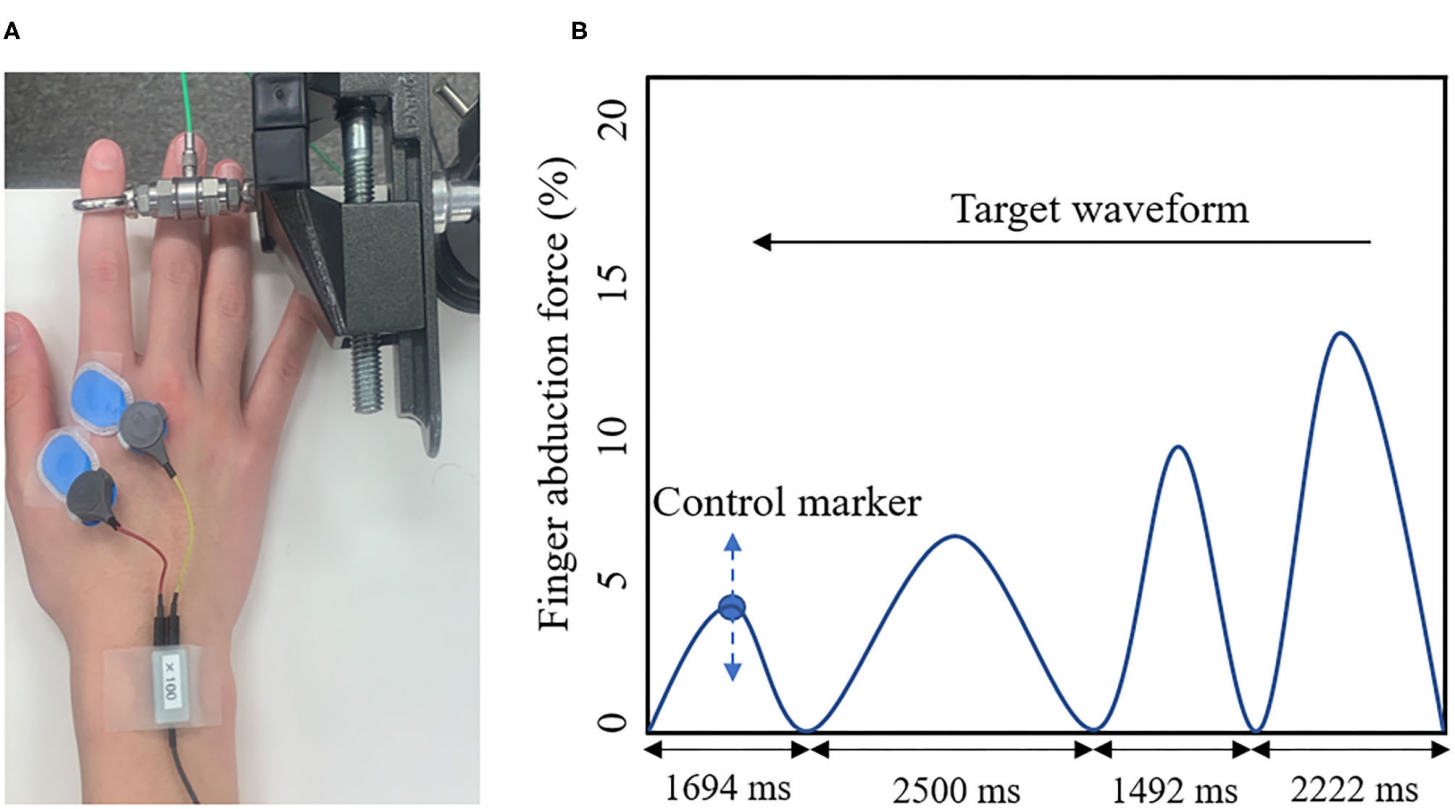
**(A)** Visual Tracking Task. The tension gauge was fixed to the right index finger, and the abduction tension was measured. **(B)** Target waveform. The blue circle indicates the manipulation markers that moved up and down according to the subject's abduction tension. The blue line shows the target waveform. The target waveform was set to 0–15% of the subject's maximum abduction force and was presented as moving from right to left on the monitor. The subject adjusted the abduction force of the indicated finger so that the manipulated marker accurately overlapped with the target waveform on the monitor.

The present study used a visual tracking task as the motor learning task (Figure 2). A force gauge was fixated to the subject's right index finger. The participant then performed the isometric abduction movements so that the marker, which moved up and down depending on the abduction force of the index finger produced by the subject, was accurately aligned with the waveforms, which were displayed on a laptop screen as flowing from the right to the left. The width of the waveforms was set to 2,500, 2,222, 2,083, 1,694, and 1,492 ms (Miyaguchi et al., 2018). The movement intensity was set to five levels, ranging from 0 to 15% (5, 8, 10, 12, and 15%) of the subject's maximum index finger abduction force (Miyaguchi et al., 2018). Each block was presented randomly in a total of 50 waveforms and took 50 s.

### Experimental Procedure

**Figure 3:**
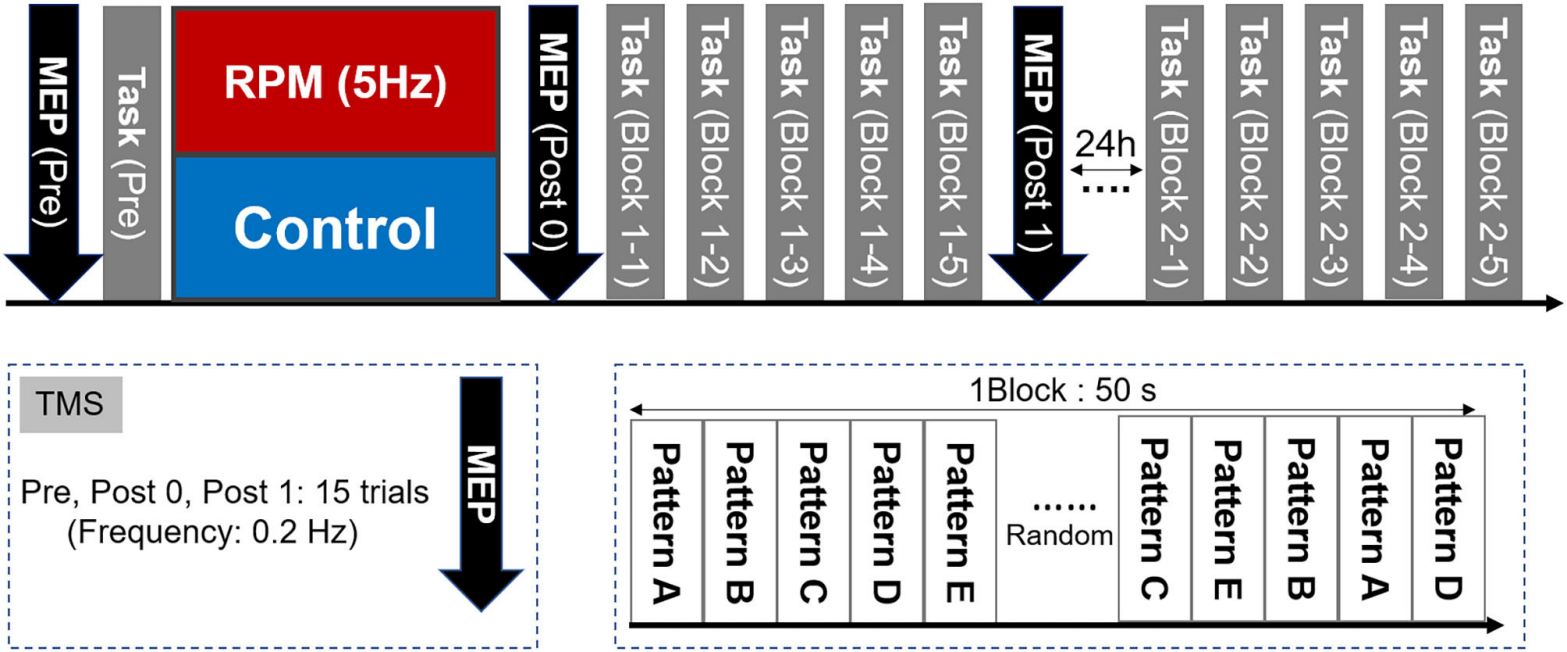
Experimental Protocol. First, we measured the MEP as a baseline value and evaluated the motor performance in the visual tracking task. The MEP was then measured again after the intervention of either the RPM condition or the Control condition. Subsequently, five visual tracking tasks were performed as a motor learning task. A total of 50 waveforms were presented randomly per block. One block lasted 50 s. A 10 s break was provided between each block, to avoid fatigue. After the motor learning task, the MEP was measured again. On the following day (24 h later), five blocks of the visual tracking task were performed in the same way as that used on the 1st day, to assess motor performance retention.

The experimental design is shown in Figure 3. First, each subject determined the site and intensity of TMS stimulation, for MEP measurement and the visual tracking task (Pre). Subsequently, each condition was subjected to an intervention (RPM condition or Control condition). We performed the MEP measurement (Post0) and 5 blocks of the visual tracking task immediately after the intervention (each Block 1-1, 1-2, 1-3, 1-4, and 1-5). The visual tracking task was performed with a 10 s interval between each block to avoid muscle fatigue. Subsequently, MEP was measured again (Post1). We also performed the 5-block visual tracking tasks on the next day (24 h later), to evaluate the retention of motor performance (each Block 2-1, 2-2, 2-3, 2-4, and 2-5).

### Data Analysis

The MEP amplitude value was used as an index of M1 excitability, and the average peak-to-peak values of the MEP amplitude of 15 waveforms measured at each time were calculated (Pre, Post 0, and Post 1) (Rosenkranz et al., 2014; Vaseghi et al., 2015). MEP amplitudes below 50 μV were excluded from the mean values. Moreover, we calculated the MEP ratio before and after the intervention (Post1/Pre) and the MEP ratio before and after motor learning task (Post1/Post0) as the index of the change in M1 excitability induced by the intervention and motor learning task under each condition. As the index of homeostatic plasticity of each intervention and motor learning task, the value of the MEP ratio before and after motor learning was divided by the MEP ratio before and after the intervention [(Post1/Post0)/(Post0/Pre)] (Lang et al., 2004; Siebner et al., 2004), according to the reports of Lang et al. (2004) and Siebner et al. (2004).

As the error value, we calculated the absolute value of the difference between the abduction force of the finger and the target waveform. We calculated the error value of each block normalized by the average error value of all blocks as the task error. In addition, as an index of motor learning efficiency, we calculated the change rate of task error on the 1st day by normalizing the task error of Block 1-5 with the task error of Pre. Similarly, we calculated the change rate of task error on the 2nd day by normalizing the task error of Block 2-5 with the task error of Block 2-1.

### Statistical Analysis

The normal distribution of the data was assessed using the Shapiro–Wilk test. A mixed analysis of variance (ANOVA) was used to compare the change in MEP amplitudes for CONDITION (RPM and Control condition) and TIME (Pre, Post0, and Post1). Task error was also analyzed using a mixed ANOVA for CONDITION (RPM and Control condition) and TIME (Pre, Block 1-1, Block 1-2, Block 1-3, Block 1-4, Block 1-5, Block 2-1, Block 2-2, Block 2-3, Block 2-4, and Block 2-5). Mauchly's test of sphericity was used to analyze the sphericity of the data obtained in each experiment. When Mauchly's test of sphericity could not be adopted, the Greenhouse–Geisser correction statistic was used. When a significant main effect or interaction was found, Tukey's HSD test was used for *post-hoc* comparisons. The correlation between MEP ratio and motor learning efficiency was assessed using Spearman's test. Statistical significance was set at *P* < 0.05 for all tests.

## Results

### MEP Amplitude

**Figure 4:**
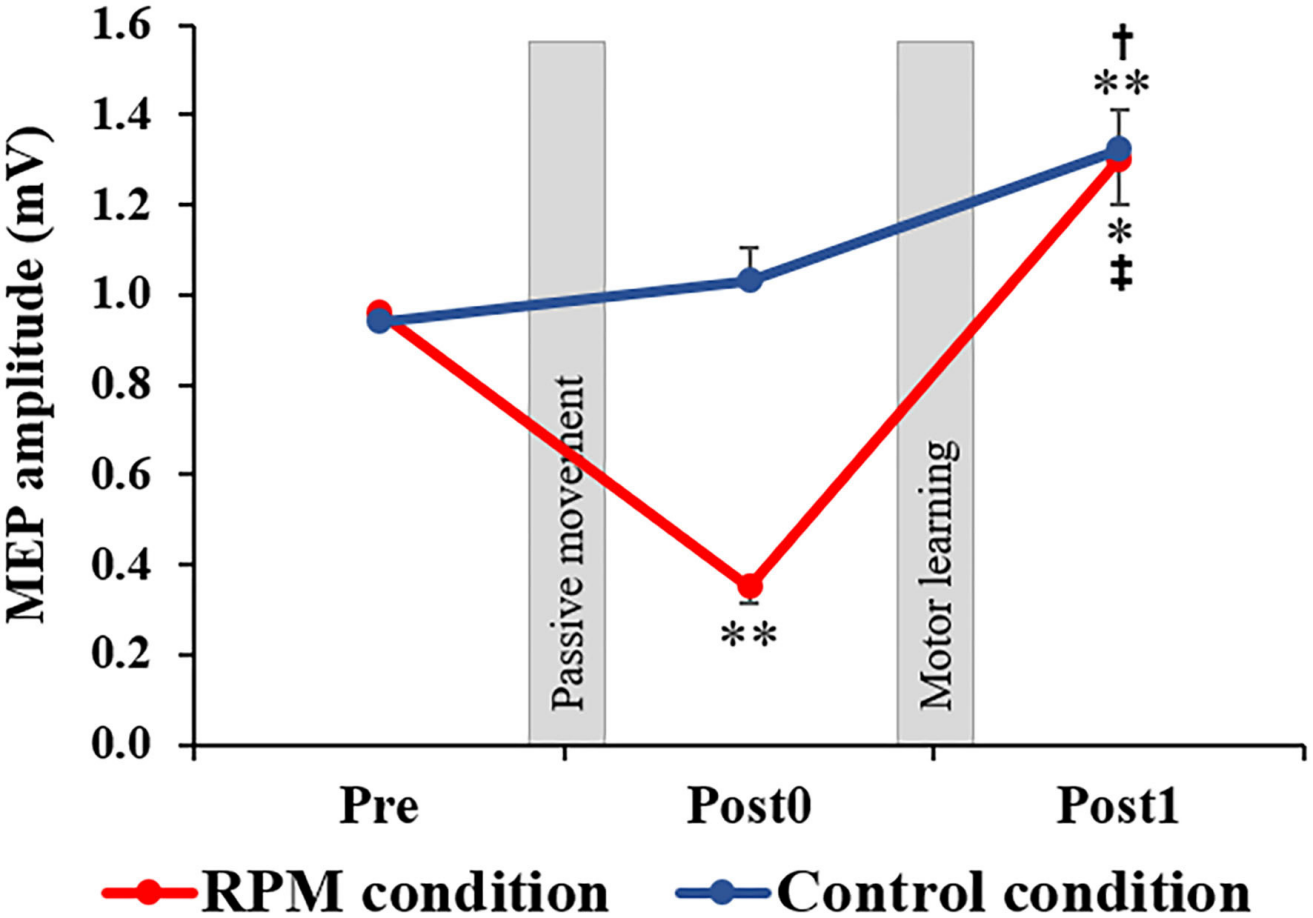
Changes in MEP amplitude in each condition. The red line indicates the average of the MEP amplitudes in the RPM condition. The blue line shows the average of the MEP amplitudes in the Control condition. The error bars indicate the standard error (SE). After each intervention, the MEP amplitude was significantly reduced in the RPM condition, whereas no change was observed in the Control condition. After the motor learning task, the MEP amplitude of the RPM condition increased to the same level as that of the Control condition. *P < 0.05 (vs. Pre); **P < 0.01 (vs. Pre). ^†^*P* < 0.05 (vs. Post0); ^‡^*P* < 0.01 (vs. Post0).

Figure 4 shows the MEP amplitudes before and after each conditional intervention. Mixed ANOVA was used for the CONDITION and TIME factors to compare the MEP amplitudes. The results showed the main effects of the CONDITION factor [*F*_(1, 38)_ = 10.694, *P* < 0.001, η^2^ = 0.220] and the TIME factor [*F*_(1.336, 50.751)_ = 33.388, *P* < 0.001, η^2^ = 0.468]. A CONDITION × TIME interaction was also observed [F_(2,76)_ = 13.193, *P* < 0.001, η^2^ = 0.258]. The *post-hoc* test results showed that there was no significant change of MEP between Pre and Post0 under the Control condition (*P* = 0.693), whereas a significant MEP increase was observed in Post1 (*p* = 0.003). Moreover, a significant MEP increase was observed in Post1 compared with Post0 under the Control condition (*P* = 0.030). Conversely, a significant MEP decrease at Post0 (*P* = 0.001) and a significant MEP increase at Post1 (*P* = 0.013) were observed under the RPM condition. Furthermore, a significant MEP increase was observed at Post1 compared with Post0 under the RPM condition (*P* = 0.001).

### Motor Learning

**Figure 5:**
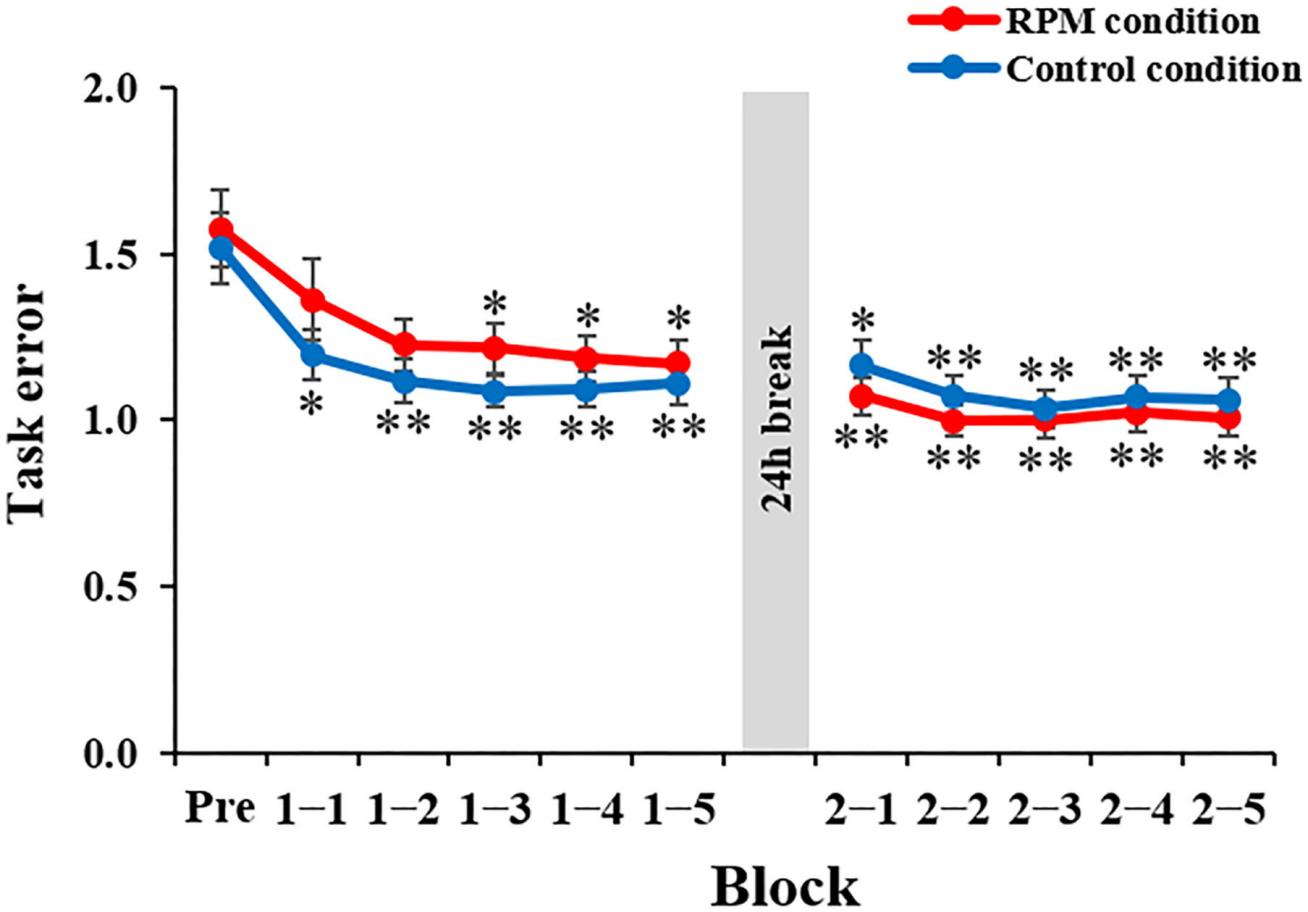
Change in the task error in each condition. The red line shows the average task error under RPM conditions. The blue line indicates the average task error of the control condition. The error bars indicate the standard error (SE). *P < 0.05 (vs. Pre); **P < 0.01 (vs. Pre).

Figure 5 depicts the task error changes under each condition. Mixed ANOVA was used for the CONDITION and TIME factors, for comparison. The results showed a CONDITION × TIME interaction [*F*_(10,380)_ = 3.669, *P* < 0.001, η^2^ = 0.089] and the main effect of the TIME factor [*F*_(3.068, 116.582)_ = 40.007, *P* < 0.001, η^2^ = 0.513], but not a main effect of the CONDITION factor [*F*_(1,38)_ = 0.091, *P* = 0.765, η^2^ = 0.002]. The results of the *post-hoc* test showed that there was no significant decrease in the task error from Block 1-1 to Block 2-5 under the Control condition compared with the Pre-condition (Bock 1-1: *P* < 0.05; Block 1-2 to Block 2-5: *P* < 0.01). Conversely, there was a significant decrease in the task error from Block 1-3 to Block 2-5 under the RPM condition compared with the Pre-condition (Block 1-3 to Block 2-1: *P* < 0.05; Block 2-2 to Block 2-4: *P* < 0.01), but no significant change in Block 1-1 and Block 1-2 (all *p* > 0.05). These results showed that the RPM condition induced a decrease in the motor learning ability immediately after the intervention. On the following day, we observed no significant change in the task error from Block 2-2 to Block 2-5 compared with Block 2-1 under both conditions (all *P* > 0.5).

### The Correlation of MEP and Motor Learning

**Figure 6:**
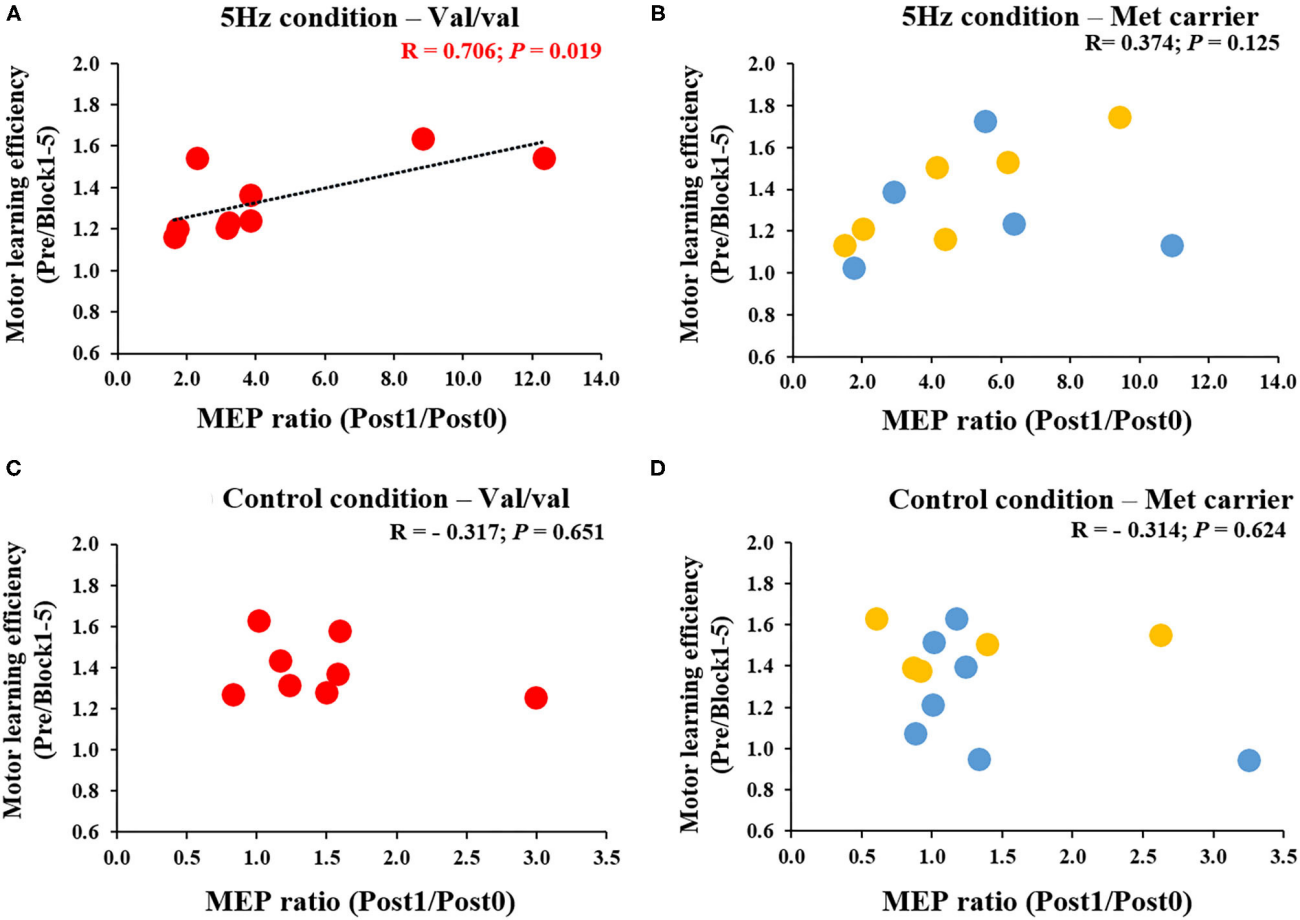
**(A–D)** Correlation between MEP ratio (Post1/Post0) and motor learning efficiency in carriers of each of the genetic polymorphisms of *BDNF*. The red circles indicate the Val/Val genotype, whereas the blue circles indicate the Val/Met genotype, and the yellow circles indicate the Met/Met genotype. There was a significant correlation between MEP ratio and motor learning efficiency before and after motor learning task in the RPM condition only for Val/Val genotype carriers (*r* = 0.706; *P* = 0.019). These results indicate that in the Val/Val form in the RPM condition, corticospinal excitability was more likely to be increased in individuals whose task errors were reduced by motor learning task.

**Figure 7:**
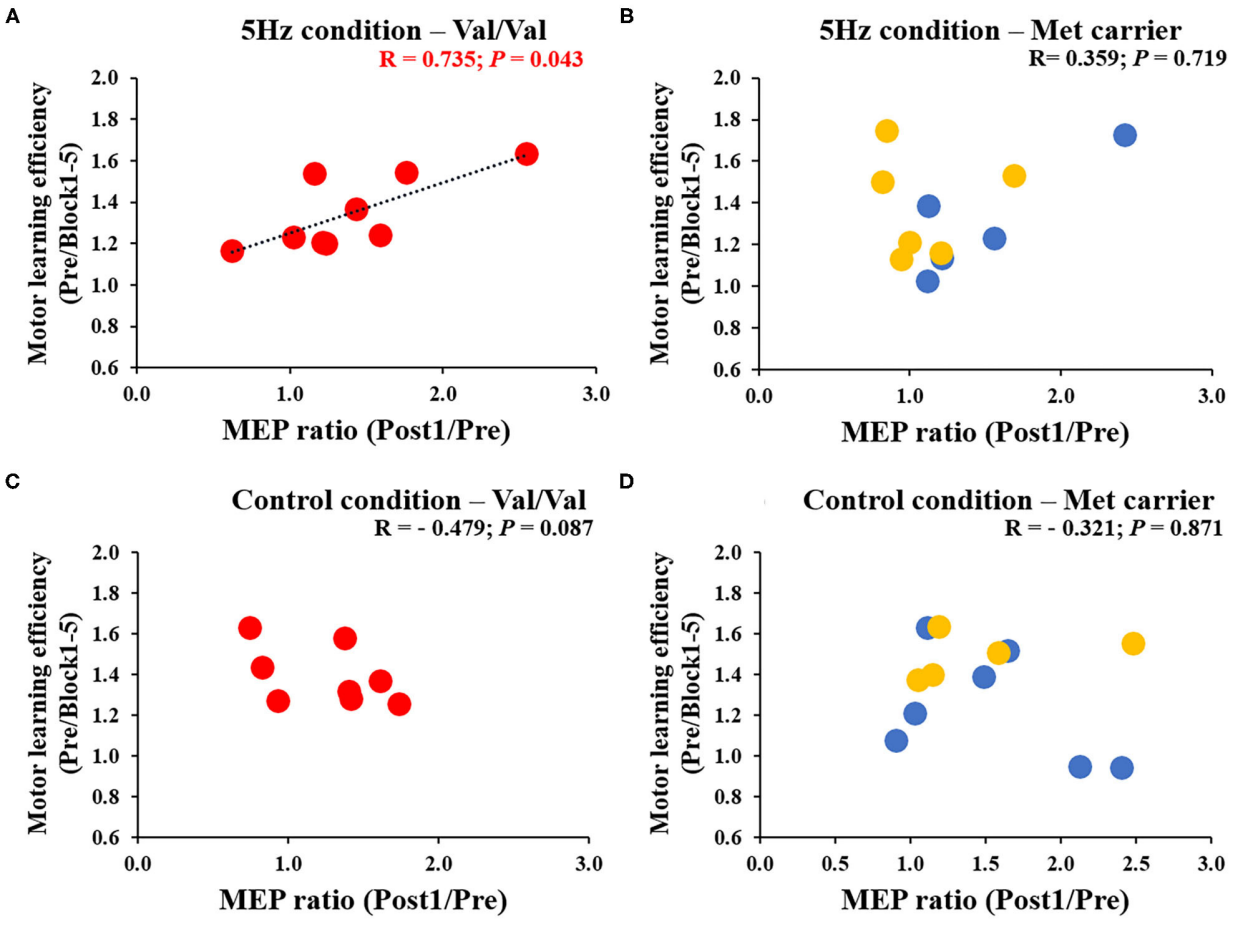
**(A–D)** Correlation between MEP ratio (Post1/Pre) and motor learning efficiency to Pre for each genetic polymorphism of *BDNF*. The red circles indicate the Val/Val, the blue circles are the Val/Met, and the yellow circles are the Met/Met genotypes. In the RPM condition alone, there was a significant correlation between MEP ratio and motor learning efficiency for the Val/Val genotype (*r* = 0.735; *P* = 0.043). These results showed that in the case of the Val/Val genotype in the RPM condition, corticospinal excitability was increased in individuals whose task errors were reduced by motor learning task.

Figure 6 depicts the correlation between the MEP ratio (Post1/Post0) and the motor learning efficiency (Pre/Block 1-5) on the 1st day and the *BDNF* gene polymorphism. The RPM condition yielded a correlation between the MEP ratio and the motor learning efficiency in Val/Val carriers before and after exercise practice (*r* = 0.706, *P* = 0.019; Figure 6A), but not in Met carriers (*P* = 0.125; Figure 6B). This result showed that in carriers of the Val/Val genotype, MEP increased as the task error was decreased by motor learning task. The control condition showed no correlations in both groups, i.e., Val/Val and Met carriers (Val/Val: *P* = 0.651; Met carriers: *P* = 0.624; Figures 6C,D). Similarly, after the motor learning task from Pre under the RPM condition, there was a significant positive correlation between the MEP ratio (Post1/Pre) and the motor learning efficiency in Val/Val carriers (*r* = 0.735, *P* = 0.043; Figure 7A), but not in Met carriers (*P* = 0.719; Figure 7B). In addition, under the Control condition, there was no correlation in both groups (Val/Val: *P* = 0.087; Met carriers: *P* = 0.871; Figures 7C,D).

### Correlation Between Homeostatic Plasticity and Motor Learning

**Figure 8:**
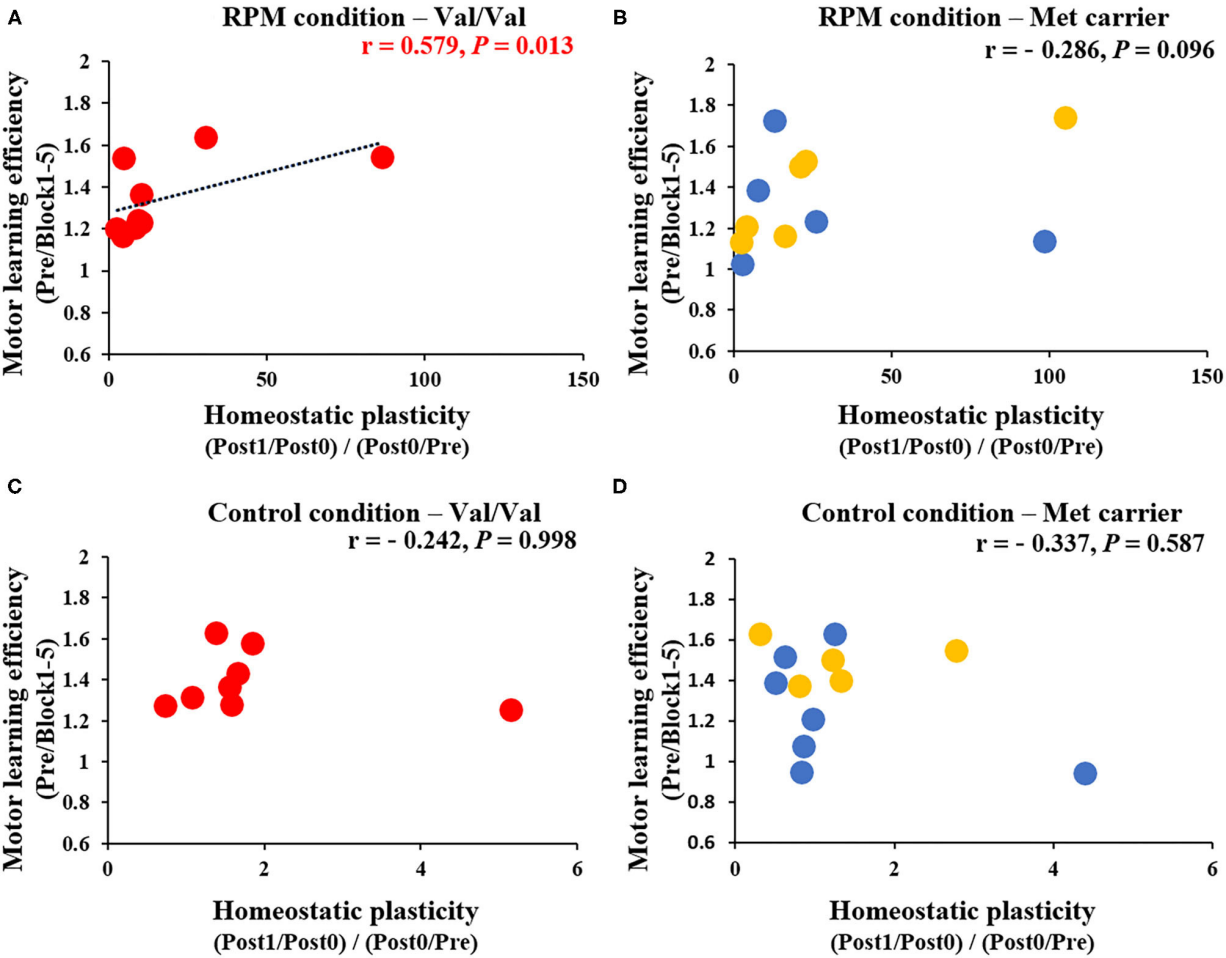
**(A–D)** Correlation between homeostatic plasticity and motor learning efficiency for each genetic polymorphism. The red circles are the Val/Val, the blue circles are the Val/Met, and the yellow circles are the Met/Met genotypes. A significant correlation between homeostatic plasticity and motor learning efficiency was found for the Val/Val genotype in the RPM condition alone (*r* = 0.579; *P* = 0.013). These results suggest that in carriers of the Val/Val genotype, the motor learning rate was more likely to be higher in subjects who exhibited homeostatic-plasticity-like plastic changes in the RPM condition.

Figure 8 shows the correlation between MEP variability [(Post1/Post0)/(Post0/Pre)] and Day 1 motor learning efficiency (Pre/Block 1-5) induced by the passive movement intervention and motor learning task. After classifying results and examining them according to *BDNF* gene polymorphisms, a significant positive correlation was noted between MEP variability and the motor learning efficiency induced by the passive movement intervention and motor learning task in Val/Val carriers (*r* = 0.579, *P* = 0.013; Figure 8A), but not in Met carriers (*P* = 0.096; Figure 8B). These results showed that the higher MEP variability caused by the passive movement intervention and motor learning task lowered the error rate in Val/Val carriers. Conversely, under the Control condition, there was no relationship between MEP variability and motor learning efficiency in both Val/Val and Met carriers induced by passive movement intervention and motor learning task (all *P* > 0.05; Figures 8C,D).

## Discussion

In the present study, we examined the manner in which an RPM intervention delivered before motor learning tasks impacts the subsequent motor learning ability. The results indicated that, although the motor learning ability transiently declined after the 5 Hz RPM, the motor performance finally improved to the same level as that detected in the Control condition; moreover, the corticospinal excitability was also increased. Furthermore, it was shown that, in the subjects with the Val/Val genotype, there was a relationship between the increased corticospinal excitability and the improved motor performance afforded by motor learning task after RPM.

The present study showed that the MEP amplitude declined transiently after 5 Hz RPM. Previous studies have also reported that MEP amplitude transiently decreased after RPM (Brasil-Neto et al., 1994; Teo et al., 2012b; Miyaguchi et al., 2013, 2016; Sasaki et al., 2017; Onishi, 2018). In the present study, we used the 5 Hz RPM intervention, which also decreased M1 excitability for 15 min after the intervention, as shown in a previous study (Sasaki et al., 2017); moreover, the results of this study also supported this hypothesis.

In the present study, subjects performed a visual tracking task, which may increase the corticospinal excitability, while experiencing decreased corticospinal excitability. While we had predicted that homeostatic plasticity would cause a marked increase in the excitability of the corticospinal tract, it was equivalent to that of the Control condition. In previous studies, the effect of the rTMS intervention depended on the M1 excitability before the intervention (Lang et al., 2004; Siebner et al., 2004; Quartarone et al., 2005; Bocci et al., 2014). Thus, similar to that reported by previous studies (Siebner et al., 2004; Ziemann et al., 2004; Ziemann and Siebner, 2008), it is assumed that homeostatic-plasticity-like changes would occur by performing a motor learning task that increased the M1 excitability after a 5 Hz RPM intervention that decreased M1 excitability. However, the present study showed that the MEP recorded after motor learning task under the RPM condition was similar to that observed under the Control condition and did not increase more than that of the control group. It appears that the time interval between the two interventions may have affected this process (Müller-Dahlhaus and Ziemann, 2015). Previous studies have shown that different time intervals between the two interventions result in different changes in plasticity (Jung and Ziemann, 2009; Fricke et al., 2011). Therefore, it is supposed that the time interval between the two interventions might affect the plastic changes; however, the mechanism underlying this phenomenon has not been elucidated in detail (Jung and Ziemann, 2009; Monte-Silva et al., 2010, 2013; Müller-Dahlhaus et al., 2015). Therefore, in the present study, different effects may be obtained by varying the time interval between the RPM intervention and the motor learning task. Thus, going forward, we would like to investigate the details of the effect of the time interval between the two interventions.

Previous studies have shown that the motor learning ability is improved by performing motor learning task during periods of reduced M1 excitability (Jung and Ziemann, 2009); however, the present study did not confirm those findings. The difference in the methods of the interventions used as preconditioning may explain this discrepancy. The reported intervention methods used for preconditioning include repetitive transcranial magnetic stimulation (rTMS), transcranial direct current stimulation (tDCS), and PAS (Lang et al., 2004; Siebner et al., 2004; Ziemann et al., 2004; Gentner et al., 2008; Jung and Ziemann, 2009; Siebner, 2010; Fricke et al., 2011; Schambra et al., 2011). Therefore, its impact on motor learning ability may vary according to the intervention methods used for preconditioning (Karabanov et al., 2015; Lopez-Alonso et al., 2018). Furthermore, we can consider that the results of the present study were also influenced by the difference in the motor learning tasks used after the preconditioning intervention. Previous studies used motor learning tasks, including the thumb tapping task and the sequence reaction time task (SRTT), after preconditioning for the M1, to show that the learning ability in the thumb tapping task improved (Jung and Ziemann, 2009), while the learning ability in the SRTT task remained unchanged (Kuo et al., 2008). The present study used a visual tracking task as a motor learning task, and this difference in motor learning tasks may have affected our results compared with those of previous studies.

In the present study, motor learning ability was transiently decreased in the early stages of motor learning task, although it finally became similar to that observed in the control condition. Previous studies have reported that the M1 excitability plays an important role in motor learning (Muellbacher et al., 2000, 2001; Stagg et al., 2011a; Kolasinski et al., 2019). Reportedly, motor learning is inhibited by reduced M1 excitability (Kuo et al., 2008; Stagg et al., 2011b). Thus, the 5 Hz RPM used in the present study may also be responsible for the transient decrease in motor performance observed immediately after the RPM intervention because of the reduction of M1 excitability.

Here, *BDNF* gene polymorphisms also affected the correlation between motor learning and increased MEP amplitude. We found that Val/Val genotype carriers alone showed a positive correlation between MEP variability and motor learning rates after the RPM intervention and motor learning. The Val/Val genotype carriers secrete more BDNF compared with the Met carriers, and this genotype is prone to cause plastic changes in nerve cells (Kleim et al., 2006; Fritsch et al., 2010; Cirillo et al., 2012). The Val/Val genotype is also more prone to the plastic changes in the M1 that are associated with motor learning task and to improve motor performance (Fritsch et al., 2010; McHughen et al., 2010). Moreover, it is obvious that the Val/Val genotype is undoubtedly more prone to homeostatic plasticity than are the Val/Met and Met/Met genotypes (Cheeran et al., 2008). In light of these previous studies, the association detected between homeostatic plasticity and the motor learning ability in the present study may have been caused by the fact that, in subjects with the Val/Val genotype, homeostatic plasticity is prone to be caused by the RPM intervention and motor learning task, with motor learning therefore improving. However, this study had several limitations, including the inadequate sample size of the Val/Met and Met/Met genotype groups, as well as the inability to perform comparisons among the three groups of Val/Val, Val/Met, and Met/Met genotypes. In the future, this topic warrants study in greater detail using a larger sample size.

## Conclusions

In the present study, we showed that, although the motor learning task performed after passive motor intervention does not affect the M1 excitatory changes and motor learning ability, the effects differed according to the genetic polymorphism of the subjects. In particular, we demonstrated that, in Val/Val genotype carriers, the greater M1 excitatory change induced by the passive exercise intervention and motor exercise led to a greater improvement in motor learning ability. It was also suggested that this might be associated with homeostatic-plasticity-like changes occurring in the M1.

## Data Availability Statement

The raw data supporting the conclusions of this article will be made available by the authors, without undue reservation.

## Ethics Statement

The studies involving human participants were reviewed and approved by Ethics Committee at Niigata University of Health and Welfare. The patients/participants provided their written informed consent to participate in this study.

## Author Contributions

HO and MP conceived the study, designed the experiments, and wrote the manuscript. MP, HW, and SM performed the experiments and statistical analysis. HO, NO, and KS performed data interpretation. SM, HW, KS, and NO helped in writing the manuscript. All authors have read and approved the final manuscript.

## Conflict of Interest

The authors declare that the research was conducted in the absence of any commercial or financial relationships that could be construed as a potential conflict of interest.

## Abbreviations

ANOVA: Analysis of variance
BDNF: Brain-derived neurotrophic factor
FDI: First dorsal interosseous
MEP: Motor evoked potential
MRI: Magnetic resonance imaging
M1: Primary motor cortex
PAS: Paired association stimulation
PED: Post-exercise depression
RPM: Repetitive passive movement
SE: Standard error
SICI: Short interval intracortical inhibition
SRTT: Sequence reaction time task
TMS: Transcranial magnetic stimulation.
